# Dispersion and ground deposition of radioactive material according to airflow patterns for enhancing the preparedness to N/R emergencies

**DOI:** 10.1016/j.jenvrad.2020.106178

**Published:** 2020-05

**Authors:** M.A. Hernández-Ceballos, M. Sangiorgi, B. García-Puerta, M. Montero, C. Trueba

**Affiliations:** a European Commission, Joint Research Centre, Ispra, Italy; b Department of Environment, Radiation Protection of Public and Environment Unit, Research Centre for Energy, Environment and Technology (CIEMAT), Spain

## Abstract

The intent of minimizing the impact of the large amount of radioactive material potentially released into the atmosphere in a nuclear event implies preparedness activities. In the early phase and in absence of field observations, countermeasures would largely rely on a previous characterization of the transport and dispersion of radioactive particles and the potential levels of radioactive contamination. This study presents a methodology to estimate the atmospheric transport, dispersion and ground deposition patterns of radioactive particles. The methodology starts identifying the main airflow directions by means of the air mass trajectories calculated by the HYSPLIT model, and, secondly, the dispersion and the ground deposition characteristics associated with each airflow pattern by running the RIMPUFF atmospheric dispersion model. From the basis of these results, different products can be obtained, such as the most probable transport direction, spatial probability distribution of deposition and the geographical probability distribution of deposition above certain predefined threshold. The method is trained on the HYSPLIT trajectories and RIMPUFF simulations during five consecutive years (2012–2016) at the Almaraz Nuclear Power Plant, in Spain. 3644 forward air mass trajectories were calculated (at 00 and 12 UTC, and with duration of 36 h). Eight airflow patterns were identified, and within each pattern, the persistent days, i.e. those days in which trajectories at 00 and 12 UTC grouped into the same airflow pattern, were extracted to simulate the atmospheric dispersion and ground deposition following a hypothetical ISLOCA accident sequence of 35 h. In total, 833 simulations were carried out, in which ground contamination was estimated at cell level on a non-homogeneous geographical grid spacing up to 800 km from Almaraz. The corresponding outcomes show a large variability in the area covered and in deposition values between airflow patterns, which provide comprehensive and oriented information and resources to decision makers to emergency management.

## Highlights

• Methodology to enhance the preparedness to N/R emergencies is presented.
• Modelling results are used to estimate the most vulnerable areas to radioactive contamination.
• The HYSPLIT forward trajectories and RIMPUFF dispersion simulations are combined.
• The method is trained at the Almaraz NPP for five years (2012–2016).

## 1 Introduction

The uncontrolled release of radioactivity to the atmosphere can cause significant radioactive exposure (e.g. Chernobyl and Fukushima nuclear accidents (Steinhauser et al., 2014), becoming a threat to public health and environment and giving rise to important socioeconomic consequences. The importance to develop and/or strengthen in advance an emergency preparedness program to face nuclear or radiological (hereafter N/R) emergencies is well recognized (e.g. Andersson et al., 2018) and it is also considered in the latest Basic Safety Standards (European Union, 2013) for the various types of emergencies identified under different potential emergency exposure situations. The outcomes of this program must provide comprehensive and oriented information and resources to decision makers to emergency managements.

During the early phase of the emergency, quick decisions must be taken to establish protective actions, such as sheltering and evacuation for the potentially affected population and environment. To facilitate a preplanned strategy for protective actions, Emergency Planning Zones (EPZs) (IAEA, 2007) around nuclear power plants (NPP) are defined, being the sizes, distances and boundaries of the zones established in each country (e.g. IAEA, 2013; Ji and Qi, 2019; Hummel et al., 2020), according to their national emergency plans. However, the boundaries of these zones can be overcome depending on the type of release and the meteorological conditions (e.g. Hong et al., 2012; Lozano et al., 2011). This fact encourages the need to estimate in advance the most vulnerable areas to contamination, where decisions must be taken to establish protective actions for the potentially affected population and environment, and to include them in the elaboration of emergency preparedness programs.

Transport, dispersion and ground deposition vary according to the change in time and space of meteorological conditions, e.g. wind flow and precipitation (e.g. An et al., 2016). Hence, the success of an emergency preparedness program largely relies on adequate estimations of the atmospheric dispersion and ground deposition patterns of radioactive particles from potential sources of radioactivity (e.g. Yoshikane and Yoshimura, 2018; Choi et al., 2018). To guarantee the representativeness of the results regarding airflow patterns and deposition levels to support decision-making (e.g. to estimate the distances, the potential affected areas and the plume arrival times at critical locations) a large statistical sample is needed. Hence, there is an obligation to generate massive amount of simulations with the purpose to consider a large number of meteorological scenarios (e.g. Christoudias et al., 2014; Fawole et al., 2019). The use of meteorological and atmospheric dispersion models is needed to address this requirement (e.g. Zheng et al., 2007).

The present study proposes a methodology to provide a robust estimation of the most vulnerable areas to radioactive contamination according to the regional airflow patterns. To this purpose, the methodology, firstly, identifies general airflow patterns at a particular site, and secondly, characterizes the atmospheric dispersion and ground deposition of radioactive material associated with each wind pattern. The airflow patterns are identified by means of the air mass trajectories calculated by the Hybrid Single-Particle Lagrangian Integrated Trajectory (HYSPLIT) model (Stein et al., 2015), while the dispersion and ground deposition characteristics are estimated by using the RIsø-Mesoscale-PUFF (RIMPUFF) model system (Thykier-Nielsen et al., 1999), integrated in the JRODOS decision support system (KIT, 2017).

This methodology is trained in a case study at the Almaraz NPP (hereafter, ALM NPP) considering the HYSPLIT forward trajectories and RIMPUFF dispersion simulations of a hypothetical release scenario for all days during a five years period (2012–2016). All trajectories and dispersion simulations have been produced under the “Assessment of the Nuclear Risk in Europe - A Case Study in the Almaraz Nuclear Power Plant” (ANURE) project, which aims at developing a methodology to elaborate nuclear risk maps integrating all these local factors (García-Puerta et al., 2018). The study was led by the European Commission Directorate General Joint Research Centre (EC/DG JRC) and the research Centre for Energy, Environment and Technology (CIEMAT) in the framework of a collaboration agreement between both institutions.

This paper is organized as follows: first, the methodology is described, together with the input data and dispersion simulation set-up in section 2. Section 3 then presents and discusses the results for the case study, while in section 4 the usefulness of the methodology and results are discussed in terms of decision-making process. Finally, the conclusions are shown.

## 2 Methods

**Fig. 1:**
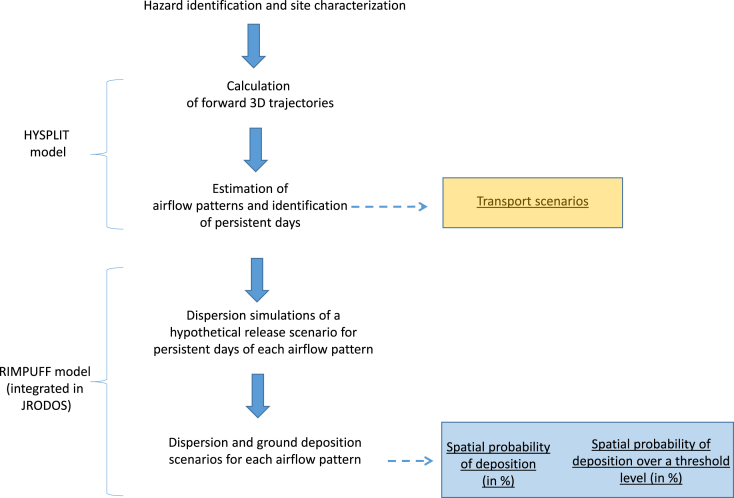
Flow diagram of the methodology combining air mass analysis and atmospheric dispersion simulations.

The basic methodological approach in the present analysis is to work with a large number of meteorological scenarios, and so, guaranteeing the largest statistical sample in determining the most probable airflow pattern, and then, the dispersion and ground deposition characteristics. Specifically, the calculation and analysis of a large number of trajectories and dispersion simulations respectively reduces the effects of individual errors of air mass trajectories in the determination of the atmospheric transport pathways (Stohl and Seibert, 1998; Fleming et al., 2012), and the model's own uncertainties (e.g. Christoudias et al., 2014). The present methodology is then based on an ensemble modeling approach, in which the ensemble members, i.e. trajectories and dispersion simulations respectively, are obtained by means of a single model that uses different initial conditions (Galmarini et al., 2004). Fig. 1 summarizes the methodology.

### 2.1 Hazard identification and site characterization

A nuclear and radiation accident is defined by the International Atomic Energy Agency (IAEA) as an event that has led to significant consequences to people, the environment or the facility. An accident in a nuclear facility can produce the release of large quantities of radioactive materials, in airborne and liquid form, to the environment. The hazard addressed is the accidental release of radioactive material from nuclear facilities to the atmosphere. Once in the atmosphere, orography features exert a very significant influence on the atmospheric dispersion of pollutants (e.g. Xie et al., 2019). Taken it into account, a prior knowledge and description of the site, e.g. main orographic features influencing the speed and direction of surface winds, and hence, the transport and dispersion of the plume in the atmosphere, is therefore required (e.g. Morales-Acuña et al., 2019).

### 2.2 Air mass trajectories: identification of airflow patterns and persistent days

Air mass trajectories are commonly applied to identify and characterize airflow patterns when compiled over multiple seasons or years (e.g. Pérez et al., 2015). The application of cluster analysis, which may be applied following different techniques (Kassomenos et al., 2010), to a database of air mass trajectories allows classifying them according to their pathways, and hence, extract the corresponding airflow patterns (e.g. Jorba et al., 2004).

The HYSPLIT model (Stein et al., 2015) is one of the most extensively used atmospheric transport and dispersion models in the atmospheric sciences community (e.g. Pirouzmand et al., 2018; Ngan et al., 2018). Trajectories are calculated with HYSPLIT using as meteorological input the GDAS-NCEP meteorological data of the NOAA/ARL (Air Resources Laboratory). These files have a temporal resolution of 3 h, spatial resolution of 1 × 1° (in latitude and longitude), and 23 vertical levels (from 1 000 to 20 hPa, with 17 of them below 300 hPa) (https://www.ready.noaa.gov/gdas1.php). The selection of GDAS files is based on the possibility to calculate kinematic 3D trajectories, which are more accurate in comparison to all the other approaches (e.g. isobaric, isentropic) (Stohl and Sibert, 1998). GDAS files include vertical velocity, and hence, the vertical motion of the trajectory can be calculated directly from the model output vertical wind velocity. These files are also recommended for driving HYSPLIT model in areas with complicated topography and diversified land-use (e.g. Su et al., 2015).

Two kinematic 3D forward trajectories per day (00 and 12 UTC) with a run time of 36 h and at an initial height of 100 m above ground level (a.g.l) are calculated and stored. The selection of 36 h forward trajectories is done because it is representative enough to identify the airflow patterns leaving from potential radioactive sources.

Trajectories are then grouped together in length and curvature by using the cluster methodology implemented in the HYSPLIT model. This method is based on variations of the total spatial variance (TSV) between the different clusters formed and the cluster spatial variance (CSV), with the purpose to minimize the differences among individual elements belonging to the same cluster and maximizes the differences among different clusters. The TSV is the sum of the CSV over all clusters. The process starts with N trajectories, and the clustering process continuously combines the two clusters that results in the minimum increase in TSV, until all trajectories are merged into one cluster. For each combination, the TSV is computed, and its variability along this process helps to identify the optimal number of clusters, which is defined as the number of clusters that best represents the air mass variability during one period (Stunder, 1996). In the present methodology, this optimal number is associated with the first variation in percentage of the TSV above 40% between two clusters in the range of the last ten combinations.

Based on the set of airflow patterns defined, the “persistent” days within each one are identified as those days on which both trajectories (at 00 and 12 UTC) belonged to the same cluster. These days are then taken as reference to characterize the dispersion and ground deposition of each airflow pattern.

### 2.3 Atmospheric dispersion and ground deposition simulations

The RIMPUFF model system (Thykier-Nielsen et al., 1999), integrated in the JRODOS decision support system (KIT, 2017), is used to simulate the atmospheric dispersion of radioactive material released following a hypothetical accident scenario in a NPP. RIMPUFF can cope well with time-dependent and spatially inhomogeneous meteorological situations, and it applies both to plain and inhomogeneous terrain on a horizontal scale of up to 50 km. This model responds to changing meteorological conditions, and simulates the time changing releases of airborne materials by sequentially releasing a series of Gaussian shaped puffs at a fixed rate on a specified grid.

Global Forecast System analysis data (GFS-ANL) produced by the National Centers for Environmental Prediction (NCEP), available at the NOMADS website (https://www.ncdc.noaa.gov/data-access/model-data/model-datasets/global-forcast-system-gfs), are used as boundary conditions of meteorological field by RIMPUFF. The temporal resolution of this meteorological data is 6 h, and the horizontal resolution is 0.5-degree (~55 km), covering the pressure interval from 1 000 to 10 hPa with 26 standard pressure levels. The possibility to get free access to these meteorological files and to extend the analysis in the future from the base of the same meteorological inputs have been the major criteria for the selection of this dataset. The JRODOS meteorological preprocessor (Andronopoulos et al., 2010) takes that input data to perform spatial interpolation to calculate meteorological information on the selected grid and it runs a “wind field model” to adjust the interpolated wind velocity components in order to assure mass conservation of the wind field.

The source term specifications vary with the type of accident and nuclear reactor (e.g. Awan et al., 2012). During a nuclear accident, the radioactive release to the atmosphere is difficult to know quickly, and it is usually incomplete and subject to many uncertainties (Tichý et al., 2018). For atmospheric dispersion and deposition studies, the characteristics of the source term, i.e. the time of release, release duration, height of release, activity released, etc., can be taken from existing studies in NPPs (e.g. NUREG/CR-7110, 2013), which provide a realistic evaluation of accident progression. In the present methodology, the source term comprises three radionuclides: ^131^I, ^90^Sr and ^137^Cs, which cover the main types of nuclide concerning dispersion and radiation properties.

Daily dispersion simulations are automatically performed using the statistical tool implemented in JRODOS. The simulation grid used has an irregular resolution. The chosen grid calculation is 800 km, which corresponds to a minimum grid cell size of 2 km around the point of release, up to 50 km, 4 km up to 100 km, 8 km up to 200 km, 16 km up to 400 km and 32 km up to 800 km. The dispersion simulations are run during enough prognosis time to ensure the total deposition in the simulation domain.

Model outputs in each cell consisted on the total ground deposition of ^131^I, ^90^Sr and ^137^Cs (Bq m^−2^) at the end of each simulation. Two analysis with respect to space are performed in order to estimate the affected areas by the radioactive deposition (location and the spatial coverage, in size and shape) over the whole simulation domain, in terms of occurrence and intensity:

- Probability of deposition: The probability of occurrence of deposits at every grid cell.
- Probability of deposition over a threshold level: The probability of occurrence of depositions above a fixed activity concentration value, e.g. threshold of protective measures, at every grid cell.

Both are used to visualize the spatial distribution of the radionuclides’ deposition according to the airflow patterns previously defined.

## 3 Case study: ALMARAZ NPP

**Fig. 2:**
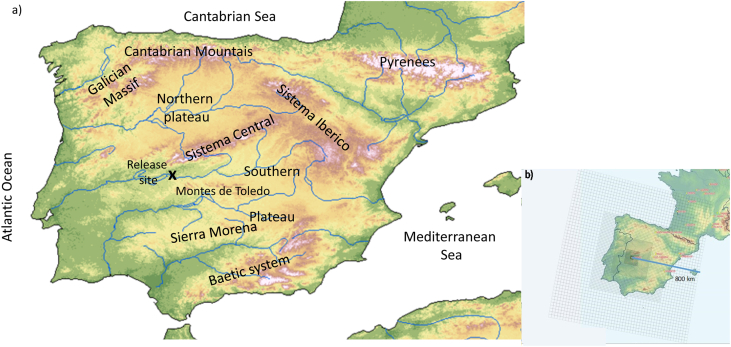
a) Area of study, and orographic features of Iberian Peninsula, and b) simulation domain. A black cross identifies the release site.

The present method as a whole has been trained at ALM NPP for five consecutive years (2012–2016) in the context of the ANURE project (García-Puerta et al., 2018). The modelling area was centered in ALM NPP (Lat = 39.80705, Long = −5.6986), at the Southwest part of the Iberian Peninsula, near the Portuguese border (Fig. 2a). This area is close to environmental protection areas (including the Monfragüe National Park and its Special Protection Area and the Environment Pastures alongside the Arrocampo area) and to cities, such as Caceres (70 km and 95 000 inhabitants), and to the metropolitan areas of Madrid, which is the forth one most populated in Europe (200 km and 7.3 million people). These are key factors to be taken into account in an emergency and post-accidental situation.

The complex orography of the Iberian Peninsula strongly influences the spatial and temporal distribution of surface winds in the selected area (Lorente-Plazas et al., 2015), which makes the area suitable for studying the dispersion patterns in complex terrain under various meteorological conditions. Close to the ALM NPP, the major mountain systems are the Sistema Central dividing the Iberian plateau into a northern and southern plateaus, the Montes de Toledo to the south, the Galician Massif in the northwestern, the Cantabrian Mountains along the northern coast and Sierra Morena forming the southern border of the southern plateau (Fig. 2).

### 3.1 Airflow patterns identification

**Fig. 3:**
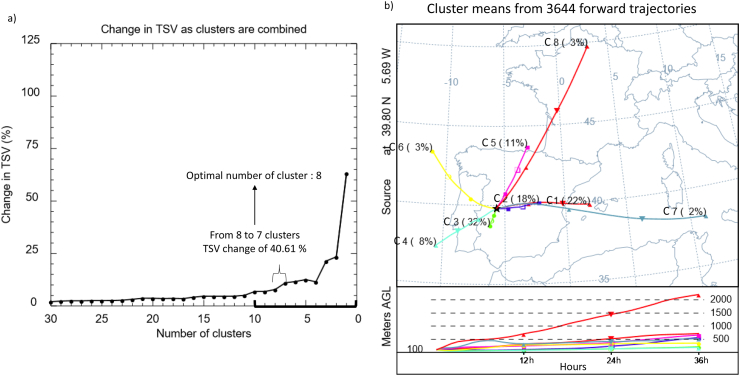
a) Variation of the total spatial variance (TSV) with the number of clusters in the final 30 clustering merge combinations. b) Mean cluster pathways (centroids). The numbers on the right in the centroids are the percent of complete trajectories occurring in that cluster and the numbers on the left represent an identification number of the centroid.

3644 forward trajectories (two per day at 00 and 12 UTC) were calculated from 2012 to 2016 at the ALM NPP. Following the methodology explained in section 2.2, Fig. 3a shows the variation (in percentages) of the TSV during the final 30 clustering merge combinations. The resulting optimal number of clusters was eight. Fig. 3b provides the mean vertical and horizontal displacement of the eight clusters formed, and the percentage of trajectories included in each cluster (frequency of occurrence) during the 2012–2016 period. It is worth mentioning that clusters, as well as trajectories, indicate an estimation of the general airflow rather than the exact pathway of an air parcel.

Fig. 3b shows five pronounced airflow patterns: to the east (C1, C2 and C7), to the north (C5 and C8), to the northwest (C6), to the southwest (C4) and nearby (C3). These results highlighted the predominance of easterly advections (42%) and nearby circulations (32%), while the rest of airflow patterns remained below the 14%. This airflow distribution, and the large percentage of westerly air masses, agree with the frequent influence of maritime air masses arriving from the Atlantic Ocean over this region (Maya-Manzano et al., 2016), which is determined by the geographical position of this site on the extreme southwest of Europe (between 30° and 60°). In this region, the presence of the semipermanent Azores high-pressure and the Icelandic low-pressure systems over the North Atlantic Ocean (Lorente-Plazas et al., 2015) determine the main arrival of westerly winds.

A total number of 1095 persistent days were identified (60% out of the total number of days) and Table 1 shows how they were grouped by airflow patterns during the 2012–2016 period. The percentage of persistent days in each one was from 70% (C3) to 31% (C7)). There was a homogeneous yearly distribution of persistent days within each cluster, especially in those more frequent (e.g. C1 – average of 51 days and C3 – average of 82 days). In any case, the number of persistent days within each pattern was large enough, thus guaranteeing a representative statistical sample of data.

**Table 1:** Number of trajectories and persistent days in each cluster, and yearly distribution of the persistent days during the 2012–2016 period.

	Eastern (E)			Northern (N)		Northwest (NW)	Southwest (SW)	Nearby (Nb)
Cluster	C1	C2	C7	C5	C8	C6	C4	C3
Number of persisntent days (%)	254 (63%)	165 (49%)	11 (31%)	102 (51%)	27 (47%)	40 (64%)	84 (57%)	412 (70%)
Yearly distribution of persistent days								
2012	51	46	0	19	2	5	13	85
2013	46	36	3	22	2	6	11	101
2014	61	38	1	26	5	4	12	86
2015	44	27	3	17	10	11	23	74
2016	52	18	4	18	8	14	25	66

### 3.2 Atmospheric dispersion and deposition simulations

**Fig. 4:**
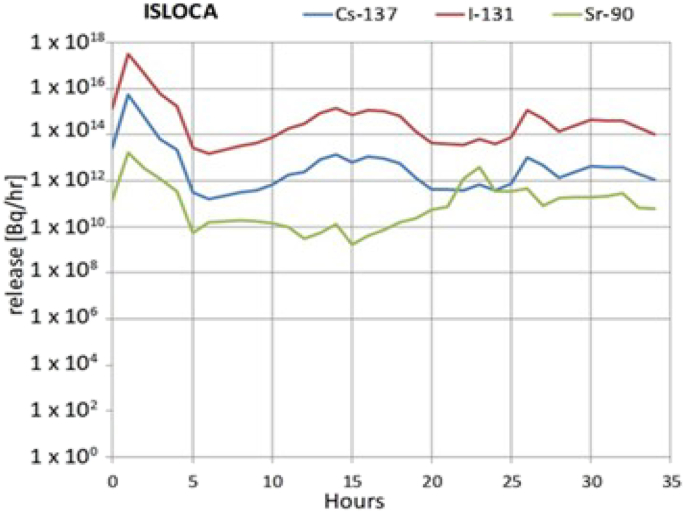
The release fractions of ^131^I, ^90^Sr and ^137^Cs during the ISLOCA sequence accidents.

An interfacing systems loss-of-coolant accident (ISLOCA), initiated by an internal event caused by an unisolated rupture of low head safety injection piping outside containment, with 35 h of offsite radionuclide release was selected as accident sequence in this case study. The corresponding source term (considering ^131^I, ^90^Sr and ^137^Cs) has been obtained from the given release fractions for the classes of halogens, alkaline earths and alkali metals (NUREG/CR-7110, 2013), and grouped on an hourly basis, to which the inventory of ^131^I, ^90^Sr and ^137^Cs of the ALM NPP, included in the JRODOS Database, was applied. Fig. 4 shows the release fractions of ^131^I, ^90^Sr and ^137^Cs during the ISLOCA accident. The stack height is 50 m and it was assumed a small heat flux so the effective release height was greater than 50 m.

For each release, the model simulated the dispersion of the plume for 83 h (35 h of release + 48 h of deposition period, section 2.3). A total number of 833 (out of 1095, 70.9% of persistent days) atmospheric dispersion simulations and the derived deposition predictions resulted valid, because of missing or corrupt meteorological files. In each cluster, the distribution of persistent days varied from 81% in C4, to 48% in C8. In spite of this variation, the number of simulations were statistically representative in all clusters during the five years period 2012–2016. Model outputs in each cell consisted on values of total ground deposition (sum of ^131^I, ^90^Sr and ^137^Cs, in Bq m^−2^) at the 83rd hour.

### 3.3 Data analysis

Here, this section presents the results according to the two treatments defined in section 2.3. For each airflow pattern and grid cell, a vector is created containing the set of total deposition values simulated at the end of each simulation (83rd hour).

#### 3.3.1 Spatial probability distribution of depositions

**Fig. 5:**
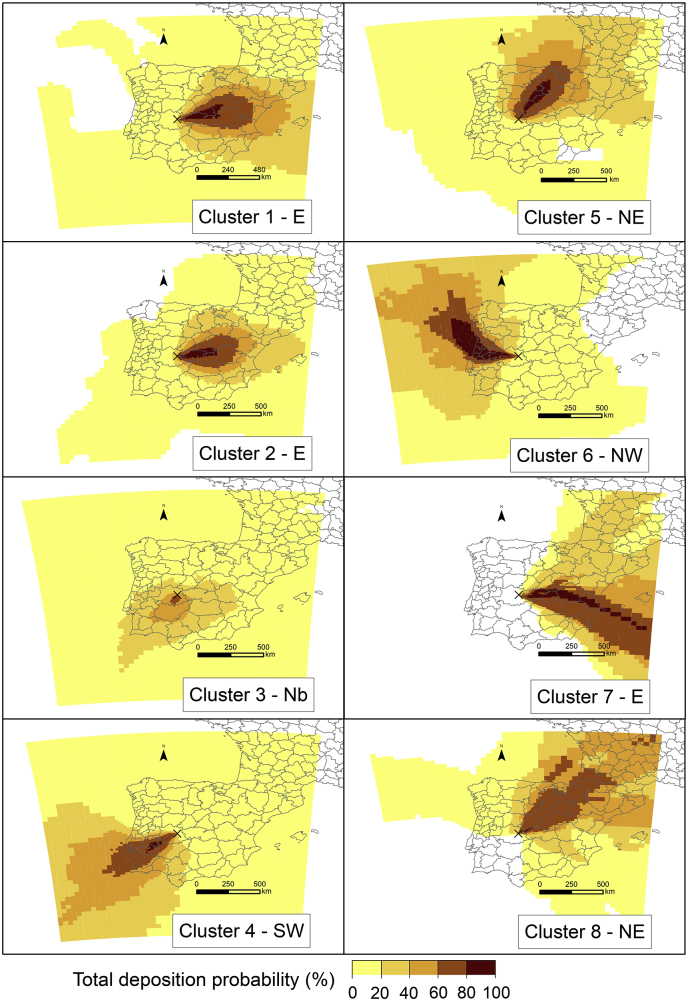
Spatial distribution of depositions for the eight airflow patterns identified in Fig. 3. The reference deposition values are taken at the end of each simulation (83 h). Projection: WGS84 (EPSG: 4326).

This analysis provides the probability of a grid cell of being affected by deposition. Fig. 5 exemplifies the spatial probability distribution of total deposition for the eight airflow patterns. Overall, the calculated distribution patterns of surface ground deposition from the release site agreed well with the transport direction marked by airflow patterns. For each airflow pattern, the deposition disperses over a larger geographic area, which is associated to changes in wind direction, and hence, in weather patterns, after the 36 h taken as reference for the cluster analysis. Nevertheless, the maximum deposition probability in each airflow pattern mostly occur along the corresponding average pathway (Fig. 3b). It highlights the small area close to the release point highly affected by deposition in C3, in contrast with those wider areas covered in C6, C7, and C8. This difference is determined by the set of different meteorological conditions influencing each airflow pattern. While for C3, stable conditions characterized by a normal field of relative high pressure in Spain (Azores high-pressure system) favouring stagnant situations and, hence, less dispersion of the plume, the other airflow patterns correspond more with synoptic scale meteorological phenomena causing larger wind speeds, and hence, dispersion processes.

#### 3.3.2 Deposition over a threshold level

**Fig. 6:**
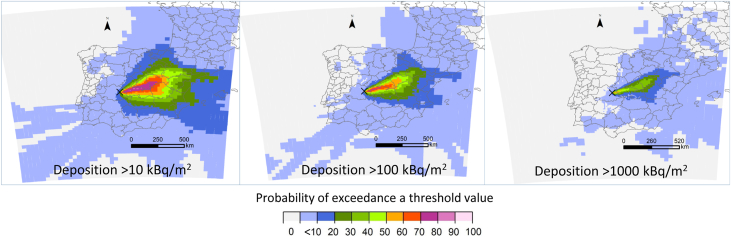
Spatial probability distribution of the eastern airflow (C1) pattern of being affected by total depositions over a) 10 kBq·m^−2^, b) 100 kBq·m^−2^, and c) 1 000 kBq·m^−2^. The reference deposition values are taken at the end of each simulation (83 h). Projection: WGS84 (EPSG: 4326).

This analysis provides the probability of a grid cell of having depositions above a given activity concentration threshold. The graphical results show the geographical distribution of activity concentration values, e.g. predefined for protective measures in early and intermediate phases of an N/R emergency. As an example, Fig. 6 shows the probability of obtaining deposition values above different contaminated segments within one of the eastern airflow patterns (C1). These levels have been taken from the Nordic Guidelines and Recommendations (NGR, 2014), which predefines five contamination levels (in logarithmic scale from slightly contaminated, less than 100 kBq·m^−2^ to extremely contaminated, over 10 000 kBq·m^−2^) for strong gamma and beta emitters together. These levels therefore provide a common starting point for the practical application of protective measures for national authorities responsible for radiation protection.

**Fig. 7:**
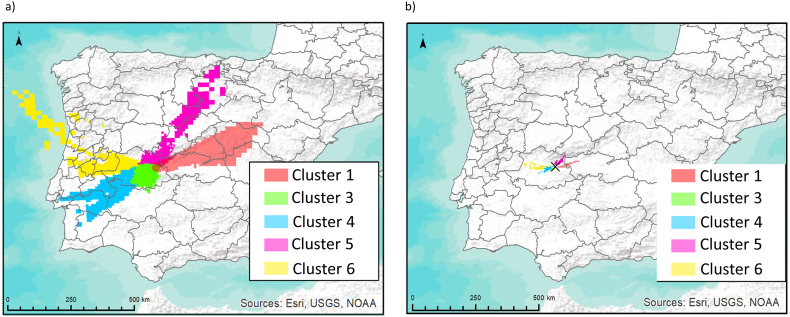
Spatial coverage of areas with a probability above a) 20% and b) 50% of being affected by depositions of ^137^Cs, ^131^I, ^90^Sr over 1 000 kBq·m^−2^ under representative airflow patterns. The reference deposition values are taken at the end of each simulation (83 h). Projection: WGS84 (EPSG: 4326).

From the basis of these results, different products can be obtained. A final plot summarizing the areas with a percentage over 20% and 50% of being decontaminated according to NGR, 2014 (activity concentration higher than 1 000 kBq·m^−2^) is shown in Fig. 7. On top of the clear differences in the geographical distributions of the areas potentially contaminated, in accordance with the airflow patterns and the probability of being affected, this figure is also an example of how the complex orography of the Iberian Peninsula (e.g. valleys, delimited by mountain barriers, plateaus, …) influence the dispersion of the plumes. The dispersion from the ALM NPP is influenced by its location along the west-east orientated part of the Tagus river basin, and the presence of the Central Mountains, which combined, cause a main dispersion along the southwest-northeast axis.

## 4 Relevance to decision-making

This section now discusses the usefulness in the context of nuclear emergency preparedness and response (NEP&R) of the method and results presented. In recent years there has been significant progress in the area of management of N/R emergencies and rehabilitation, in which the use of model forecast has taken a lead role for the organization of countermeasures and the assessment of an accident's consequences in the early phase. Given the variety of atmospheric dispersion models available, and its inherent uncertainty, there is not a model identified as the reference modelling tool to systematically support decision-making. In this sense, the use of the HYSPLIT and RIMPUFF models is an advantage of this method, as they are state-of-the-art tools, in continuous development and have been improved to become operational systems widely used and accepted to estimate the dispersion of radioactive pollutants in the atmosphere.

The present method, with results from meteorological scenarios over five years in the analysis of air mass trajectories and in the characterization of dispersion and ground deposition, fits the need of guaranteeing the largest statistical sample and hence, the representativeness of the results to support decision-making. In addition, it leaves also room to increase the number of years in the future, as the meteorological files used in both span more than 10 years of high-resolution meteorological data. The possibility to progressively increase the number of trajectories/simulations enhances the statistical significance of the estimations obtained, and hence, enriches the decision-making process.

In general, this method provides comprehensive and oriented information and resources to decision makers to emergency management in terms of the most probable wind directions, and the possible affected areas by deposition, identifying the most vulnerable areas in terms of occurrence and intensity. If an event were to happen, for instance, these results can be used to identify the corresponding airflow pattern, by accounting for the actual meteorological conditions during the accident, and to estimate in advance the corresponding ground deposition pattern and intensity and then by scaling the estimated emissions by the real release of radioactivity.

## 5 Conclusions

An approach based on dispersion modelling providing a probability of ground deposition of radioactive material according to past meteorological data has been presented in this paper. The concept and methodology consist on the analysis of air trajectories, and dispersion and deposition simulations over a period of five years (2012–2016) produced by the HYSPLIT and the RIMPUFF models respectively. The present method provides a way to cope with the present single model deficiencies, as it widely covers a large set of meteorological scenarios.

The method has been trained on the HYSPLIT trajectories (3644) and RIMPUFF dispersion simulations (833) at the Almaraz NPP, in Spain. Eight airflow patterns have been identified, and the possible affected areas by deposition associated with each one have been estimated following a hypothetical ISLOCA accident sequence of 35 h (+48 h of deposition period). The areas where the probability of exceeding an activity concentration of 1 000 kBq·m^−2^ is 20 and 50%, respectively, have been identified. These result show that the deposition results have highly depended on the airflow pattern selection, which demonstrates the large influence of wind conditions on determine the area and scale of the response in case of a nuclear event.

These results provide comprehensive and oriented information and resources to decision makers to emergency management. In the early phase, when there are not field measures yet, an overview of the trajectory of the plume and, in turn, of the potentially affected area by the deposition may be obtained. The preparedness for the most vulnerable areas can also be taken into consideration in advance and the countermeasures can be designed according to the local/regional specificities, which redounds in their higher effectiveness.

The method aims at being reproduced in European NPPs in order to estimate the geographical distribution of the activity concentrations deposited due to nuclear events with release to the atmosphere. It is necessary to remark that assumptions taken for the source term (accident type, specifications) can affect the calculation of air radionuclides concentration and ground radionuclides deposition, as well as, the use of different meteorological datasets could improve the accuracy of the results in the simulation of the dispersion, transport and deposition of nuclear material.

## Declaration of competing interest

The authors declare that they have no conflict of interest.

## References

[bib1] An H.Y., Kang Y.-H., Song S.-K., Kim Y.-K. (2016). Atmospheric dispersion characteristics of radioactive materials according to the local weather and emission conditions. J. Radiat. Prot. Res..

[bib2] Andersson K., Linde C., Magnusson S.M., Physant F. (2018). Joint Nordic nuclear research to strengthen nuclear emergency preparednessafter the Fukushima accident. J.Environ. Radioactiv.

[bib3] Andronopoulos S., Davakis E., Bartzis J.G., Kovalets I.V. (2010). RODOS meteorological pre-processor and atmospheric dispersion model DIPCOT: a model suite for radionuclides dispersion in complex terrain. Radioprotection.

[bib4] Awan S.E., Mirza N.M., Mirza S.M. (2012). Kinetic study of fission product activity released inside containment under loss of coolant transients in a typical MTR system. Appl. Radiat. Isot..

[bib5] Christoudias T., Proestos Y., Lelieveld J. (2014). Atmospheric dispersion of radioactivity from nuclear power plant accidents: global assessment and case study for the eastern mediterranean and Middle East. Energies.

[bib6] Choi G.-S., Lim J.-M., Sunny Lim K.-S., Kim K.-H., Lee J.-H. (2018). Characteristics of regional scale atmospheric dispersion around Ki-Jang research reactor using the Lagrangian Gaussian puff dispersion model. Nucl. Eng. Technol..

[bib7] EU (2013). Council Directive 2013/59/Euratom of 5 December 2013 Laying Down Basic Safety Standards for Protection against the Dangers Arising from Exposure to Ionising Radiation.

[bib8] Fawole O.G., Cai X., Abiye O., MacKenzie A.R. (2019). Dispersion of gas flaring emissions in the Niger delta: impact of prevailing meteorological conditions and flare characteristics. Environ. Pollut..

[bib9] Fleming Z.L., Monks P.S., Manning A.J. (2012). Review: untangling the influence of air-mass history in interpreting observed atmospheric composition. Atmos. Res..

[bib10] Galmarini S., Bianconi R., Klug W., Mikkelsen T., Addis R., Andronopoulos S., Astrup P., Baklanov A., Bartniki J., Bartzis J.C., Bellasio R., Bompay F., Buckley R., Bouzom M., Champion H., Amours R.D., Davakis E., Eleveld H., Geertsema G.T., Glaab H., Kollax M., Ilvonen M., Manning A., Pechinger U., Persson C., Polreich E., Potemski S., Prodanova M., Saltbones J., Slaper H., Sofev M.A., Syrakov D., Soerensen J.H., Van der Auwera L., Valkama I., Zelazny R. (2004). Ensemble dispersion forecasting, Part II: application and evaluation. Atmos. Environ..

[bib11] García Puerta B., Sangiorgi M., Hernández-Ceballos M.A., Trueba Alonso C., De Felice L., Montero Prieto M. (2018). ANURE project: towards the implementation of a nuclear risk assessment methodology. Proceedings of the Fourth NERIS Workshop “Adapting Nuclear and Radiological Emergency Preparedness, Response and Recovery to a Changing World” Held in Dublin, Ireland on 25-27 April 2018.

[bib12] Hong G.H., Hernández-Ceballos M.A., Lozano R.L., Kim Y.I., Lee H.M., Kim S.H., Yeh S.-W., Bolívar J.P., Baskaran M. (2012). Radioactivity impact in South Korea from the damaged nuclear reactors in Fukushima; an evidence of the long and short range transports. J. Radiol. Prot..

[bib13] Hummel D.W., Chouhan S., Lebel L., Morreale A.C. (2020). Radiation dose consequences of postulated limiting accidents in small modular reactors to inform emergency planning zone size requirements. Ann. Nucl. Energy.

[bib14] IAEA (2007). Arrangements for Preparedness for a Nuclear or Radiological Emergency.

[bib15] IAEA (2013). Actions to Protect the Public in an Emergency Due to Severe Conditions at a Light Water Reactor.

[bib16] Ji Y.-M., Qi M.-L. (2019). A robust optimization approach for decontamination planning of emergency planning zone: facility location and assignment plan. Soc. Econ. Plann. Sci..

[bib17] Jorba O., Pérez C., Rocadenbosch F., Baldasano J.M. (2004). Cluster Analysis of 4-day back trajectories arriving in the barcelona area, Spain, from 1997 to 2002. J. Appl. Meteorol..

[bib18] Karlsruhe Institute of Technology (KIT) (2017). JRodos Customer Guide.

[bib19] Kassomenos P., Vardoulakis S., Borge R., Lumbreras J., Papaloukas C., Karakitsios S. (2010). Comparison of statistical clustering techniques for the classification of modelled atmospheric trajectories. Theor. Appl. Climatol..

[bib20] Lorente-Plazas R., Montávez J.P., Jimenez P.A., Jerez S., Gómez-Navarro J.J., García-Valero A., Jimenez-Guerrero P. (2015). Characterization of surface winds over the iberian Peninsula. Int. J. Climatol..

[bib21] Lozano R.L., Hernández-Ceballos M.A., Adame J.A., Casas-Ruíz M., Sorribas M., San Miguel E.G., Bolívar J.P. (2011). Radioactive impact of Fukushima accident on the Iberian Peninsula: evolution and plume previous pathway. Environ. Int..

[bib22] Maya-Manzano J.A., Fernández-Rodríguez S., Smith M., Tormo-Molina R., Reynolds A.M., Silva-Palacios I., Gonzalo-Garijo A., Sadys M. (2016). Airborne Quercus pollen in SW Spain: identifying favourable conditions for atmospheric transport and potential source areas. Sci. Total Environ..

[bib23] Morales-Acuña E., Torres C.R., Linero-Cueto J.L. (2019). Surface wind characteristics over Baja California Peninsula during summer. Reg. Stud. Mar. Sci..

[bib24] Ngan F., Stein A., Finn D., Eckman R. (2018). Dispersion simulations using HYSPLIT for the sagebrush tracer experiment. Atmos. Environ..

[bib25] Nordic Guidelines and Recommendations (NGR) (2014). Protective Measures in Early and Intermediate Phases of a Nuclear or Radiological Emergency. Beredskabsstyrelsen (Denmark), Sundhedsstyrelsen (Denmark), Geislavarnir Rikisins (Iceland), Stuk (Finland), Statens Stralevern (Norway), Stral Sakerhets Myndigheten (Sweden).

[bib26] NUREG/CR-7110 (2013). State-of-the-Art Reactor. Consequence Analyses Project (SOARCA) Volume 2: Surry Integrated Analysis. Sandia National Laboratories, Albuquerque, New Mexico 87185.

[bib27] Pérez I.A., Artuso F., Mahmud M., Kulshrestha U., Sánchez M.L., García M.A. (2015). Applications of air mass trajectories. Adv. Meteorol..

[bib28] Pirouzmand A., Kowsar Z., Dehghani P. (2018). Atmospheric dispersion assessment of radioactive materials during severe accident conditions for Bushehr nuclear power plant using HYSPLIT code. Prog. Nucl. Energy.

[bib29] Stein A.F., Draxler R.R., Rolph G.D., Stunder B.J.B., Cohen M.D., Ngan F. (2015). NOAA's HYSPLIT atmospheric transport and dispersion modeling system. Bull. Am. Meteorol. Soc..

[bib30] Steinhauser G., Brandl A., Johnson T.E. (2014). Comparison of the Chernobyl and Fukushima nuclear accidents: a review of the environmental impacts. Sci. Total Environ..

[bib31] Stohl A., Seibert P. (1998). Accuracy of trajectories as determined from the conservation of meteorological tracers. Q. J. Roy. Meteorol. Soc..

[bib32] Stunder B. (1996). An assessment of the quality of forecast trajectories. J. Appl. Meteorol..

[bib33] Su L., Yan Z., Fung J.C.H., Lau A.K.H. (2015). A comparison of HYSPLIT backward trajectories generated from two GDAS datasets. Sci. Total Environ..

[bib34] Thykier-Nielsen S., Deme S., Mikkelsen T. (1999). Description of the Atmospheric Dispersion Module RIMPUFF. Technical Report RODOS (WG2)-TN(98)-02.

[bib35] Tichý O., Šmídl V., Hofman R., Evangeliou N. (2018). Source term estimation of multi-specie atmospheric release of radiation from gamma dose rates. Q. J. R. Meteorol. Soc..

[bib36] Xie J., Liao Z., Fang X., Xu X., Wang Y., Zhang Y., Liu J., Fan S., Wang B. (2019). The characteristics of hourly wind field and its impacts on air quality in the Pearl River Delta region during 2013–2017. Atmos. Res..

[bib37] Yoshikane T., Yoshimura K. (2018). Dispersion characteristics of radioactive materials estimated by wind patterns. Sci. Rep..

[bib38] Zheng D.Q., Leung J.K.C., Lee B.Y., Lam Y. (2007). Data assimilation in the atmospheric dispersion model for nuclear accident assessments. Atmos. Environ..

